# Structure-directed synthesis of bimetallic ZIF-67 LDH nanocomposites for high-performance supercapacitors†

**DOI:** 10.1039/d5ra01889g

**Published:** 2025-05-19

**Authors:** Zia Ur Rehman, Shanshan Yao, Antonio Miotello, Mouna Ben Henda, Umar Farooq, Irum Aziz, Talal M. Althagafi, Zainab M. Almarhoon, Magdi E. A. Zaki, Afaq Ullah Khan

**Affiliations:** a Institute for Advanced Materials, College of Materials Science and Engineering, Jiangsu University Zhenjiang 212013 P. R. China ziamwt1@gmail.com; b Department of Chemistry, Hazara University Mansehra-21120 Khyber Pakhtunkhwa Pakistan; c Department of Physics, University of Trento Via Sommarive 14 38123 Trento Italy; d Physics Department, College of Science, Al-Zulfi, Majmaah University Al-Majmaah 11952 Saudi Arabia; e Department of Chemistry, Abdul Wali Khan University Mardan Pakistan; f Department of Chemistry, University of Azad Jammu and Kashmir Muzaffarabad 13100 Pakistan; g Department of Physics, College of Science, Taif University Taif 21944 Saudi Arabia; h Chemistry Department, College of Science, King Saud University P. O. Box 2455 Riyadh-11451 Saudi Arabia; i Department of Chemistry, Faculty of Science, Imam Mohammad Ibn Saud Islamic University Riyadh 11623 Saudi Arabia; j School of Chemistry and Chemical Engineering, Jiangsu University 301 Xuefu Road Zhenjiang 212013 China

## Abstract

The energy storage ability of a device highly depends on the morphology of the materials used. A structure-directing agent (SDA) can be used to design materials with a specific surface morphology. Zeolite imidazole (ZIF-67) is a favorable electroactive material for energy storage devices. Here, we present a novel approach for synthesizing a ZIF-67 derived NiCo layer double hydroxide hollow surface sheet like morphology, in which potassium fluoroborate acts as a SDA. The hollow sheets possess the largest specific capacitance of 1171 F g^−1^ at 1 A g^−1^. The energy storage device composed of ZIF-67 derivatives and a carbon electrode presents a maximum energy density of 26 W h kg^−1^ at a power density of 804 W kg^−1^. The device shows good cyclic stability of 84% after 10 000 charge–discharge cycles. These outcomes reveal the promising potential of zeolite imidazole (ZIF-67)-based materials for use in next-generation energy storage devices.

## Abstract 2

The energy storage ability of a device highly depends on the morphology of the materials used.

## Graphical abstract

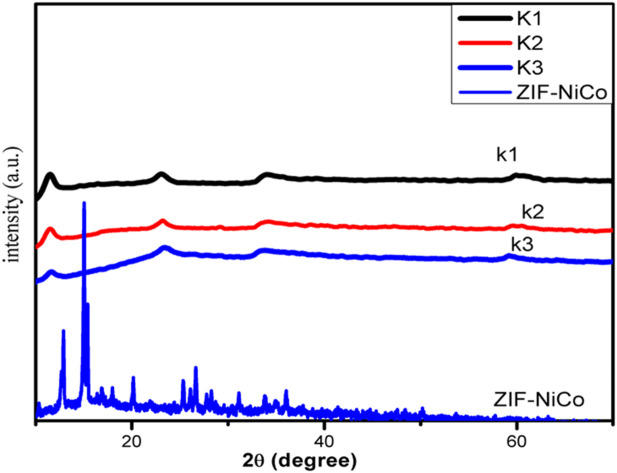

## 1 Introduction

The current depletion of conventional energy sources has stimulated the search for new and sustainable energy storage solutions, aiming to mitigate the environmental impact of traditional energy sources.^1–5^ Electrochemical supercapacitors (SCs) have emerged as promising alternatives to fuel cells and secondary batteries due to their rapid charging rates, desirable power density, long cycling life, and enhanced safety features.^6–9^ Despite their advantages, the practical application of SCs is hindered by their relatively low energy densities. Fortunately, it can be found that the energy density of an electroactive material improves with the increase of its specific capacitance and potential window.^10^ Recent research focused on enhancing the energy density of electroactive materials by increasing the specific capacitance and potential window, leading to the development of asymmetric supercapacitors (ASCs) utilizing novel electrode materials with high specific capacitance. Zeolitic imidazolate frameworks (ZIFs), a class of metal–organic framework known for their programmable compositions, morphologies, and porous structures, have attracted significant attention in SCs and electrocatalysis.^11–16^ In particular, various derivatives of ZIFs, such as layered double hydroxides (LDHs) and metal oxides/sulfides/hydroxides, have been synthesized to enhance their utility in supercapacitors by serving as templates and metal ion sources.^17–20^ Transition metal-based LDHs, due to their unique lamellar architectures that provide abundant active sites for rapid electrochemical redox reactions and high theoretical capacitances, have emerged as a popular choice in SCs^21,22^ However, the inherent drawbacks of single LDHs, including poor conductivity and severe agglomeration, limit their performance in constructing high-efficiency SCs.^23,24^ To address these challenges, the incorporation of nanomaterials with high conductivity and stability has been proposed as an effective strategy. For example, Zhao *et al.* demonstrated the preparation of CoMn-LDH@MnO_2_ nanocomposites on Ni foams as promising electrode materials for supercapacitors, exhibiting an exceptional energy density of 59.73 W h kg^−1^ at a power density of 1000.09 W kg^−1^.^25^ Wang *et al.* developed a ZIF-derived hollow Mn@NiCo-LDH nanowire, achieving a broad specific capacitance of 1574 F g^−1^ at 1 A g^−1^.^26^ Guan *et al.* synthesized Co_*x*_/NiCo nanocages with a specific capacitance of 1562 F g^−1^ at 1 A g^−1^.^20^ Similarly, Bai *et al.* prepared an *in situ* ZIF with NiCo-LDH, which exhibits a specific capacitance of 1265 F g^−1^ at 1 A g^−1^.^27^ The morphology of the nanomaterials has a large effect on the electrochemical performance, and therefore, a structure-directing agent (SDA) is used to prepared the materials for a favorable surface.^28–30^ For example, P.-Y. Lee and co-workers used NH_4_F_2_ as a SDA to synthesize Ni CoL-DH, which exhibits a specific capacitance of 1527 F g^−1^ at 1 A g^−1^.^31^ Su-Ching-Wang and co-workers prepared ZIF-67-derived NiCo-LDH using ammonium tetra fluoroborate as a SDA, which exhibits a specific capacitance of 1593 F g^−1^ at 1 Ag^−1^.^32,33^ P.-Y. Lee *et al.* synthesized perovskite ZIF-67 derivatives using ammonium fluoride as a SDA, which exhibit a specific capacitance of 490 F g^−1^ at 20 mV s^−1^.^34^

In this study, potassium tetra fluoroborate is used for the first time as a SDA to prepare NiCo layer double hydroxide. We developed a surface layer synthesis method to synthesize the bimetallic ZIF-67 (NICo-ZIF67) by simultaneously introducing Ni^2+^ and Co^2+^ and then incorporating the SDA to synthesize the bimetallic LDH. First, we synthesized the bimetallic ZIF-67 with dodecahedron morphology and then obtained the NiCo-LDH nanosheets by incorporating the SDA in an aqueous medium. The synthesized NiCo-LDH exhibits a remarkable specific capacitance of 1171 F g^−1^ at 1 A g^−1^ with a superior cyclic stability of 88% after 10 000 charge–discharge cycles.

## 2 Experimental

### 2.1. Synthesis of ZIF-NiCo nanocrystals

In the synthesis method, 0.08 g of nickel nitrate hexa hydrate and 0.52 g of cobalt nitrate hexa hydrate Co(NO_3_)_2_·6H_2_O were dissolved in 40 mL of ethanol (solution 1) and 1.3 g of 2-methyl imidazole were dissolved in 60 mL of ethanol (solution 2). Both the solutions were stirred for 10 min. After 10 min of stirring, solution 2 was transferred to solution 1 and stirred for 24 h. After the completion of the stirring time the ZIF67-NiCo nanocrystals were centrifuged and washed and finally left for drying at 60 °C overnight.

### 2.2. Synthesis of NiCo-LDH

The ZIF-67 derivative NiCo-LDH was synthesized using potassium tetra fluoroborate [KBF_4_] as a novel SDA, and the already synthesized ZIF67-NiCo bimetallic nanocrystals were used as a sacrificial template for NiCo-LDH using a sample of deionized water as a solvent. A specific amount, 0.08 g, of the synthesized ZIF-NiCo template was dispersed in 40 mL of deionized water and then different concentrations of potassium tetrafluoroborate were added to the solution, *i.e.*, 0.015 g, 0.025 g, and 0.035 g, and the solution was stirred for 3 h. After completion of the stirring time, the NiCo-LDH was centrifuged and washed, and then dried at 60 °C for 24 h. The products synthesized with different concentrations of SDA, 0.015 g, 0.025 g, and 0.035 g, are named K1, K2, and K3, respectively.

### 2.3. Assembly of the NiCo-LDH asymmetric supercapacitor

The negative electrode was fabricated following a standard procedure. A mixture of activated carbon, polyvinylidene fluoride, and acetylene black in a mass ratio of 8 : 1 : 1 was stirred together, using *N*-methyl 2-pyrrolidine as a solvent to create a slurry. A nickel foam electrode, serving as the current collector, was then immersed in this slurry for 2 min and subsequently dried for 10 h at 80 °C.

To produce the gel electrolyte, 1 g of PVP was gradually added to a 10 mL 2 M KOH aqueous solution with continuous stirring until the mixture became transparent. To synthesize the positive electrode K3, NiCo-LDH, activated carbon and PVDF at 8 : 1 : 1 were stirred at room temperature and after the formation of slurry were coated on nickel foam. A piece of filter paper measuring 1 × 1 cm^2^ was used to separate the positive and negative electrodes. All components including the negative electrode, positive electrode, and separator were immersed in the gel electrolyte, held for 30 s, and successfully assembled into the asymmetric supercapacitor device.

### 2.4. Electrochemical analysis

In a 2 M KOH electrolyte, three-electrode systems were established for electrochemical testing. The experimental setup involved the utilization of a bipotentiostat electrochemical workstation, enabling the execution of various measurements, such as galvanostatic charge–discharge (GCD), cyclic voltammetry (CV), and electrochemical impedance spectroscopy. Within this setup, the NiCo-LDH composite acted as the working electrode. A Hg/HgO electrode functioned as the reference electrode, while a platinum foil was designated as the counter electrode. To determine the capacitance value (*C*_F_) from the GCD curve, eqn (1) was employed, where *I* represents the current density in amperes per gram (A g^−1^), Δ*t* denotes the discharge duration, and *m* signifies the mass of the active materials. Eqn (2) and (3) were utilized to compute the energy density (*E*) and power density (*P*), respectively. In these equations, Δ*V* represents the potential window in volts (V), Δ*t* signifies the discharge duration in seconds (s), and *C* denotes the specific capacitance eV.1
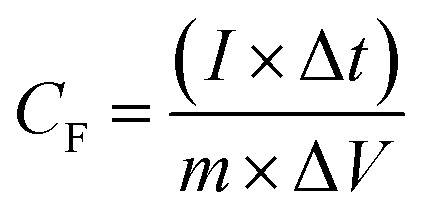
2
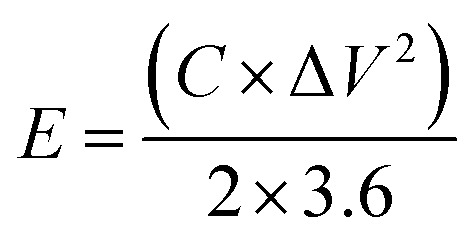
3
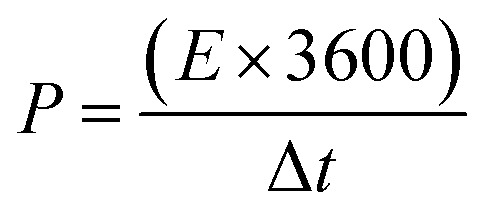


## 3 Results and discussion

**Fig. 1:**
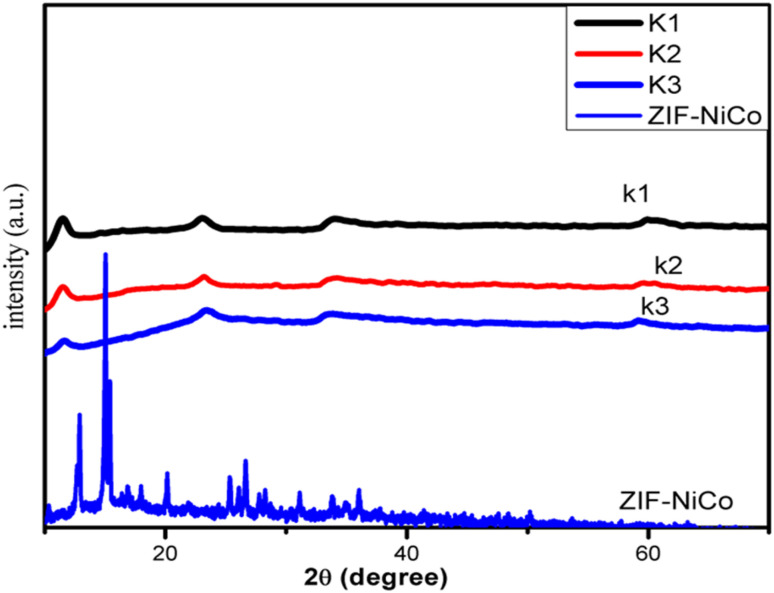
XRD patterns of ZIF-NiCo and NiCo-LDH.

**Scheme 1:**
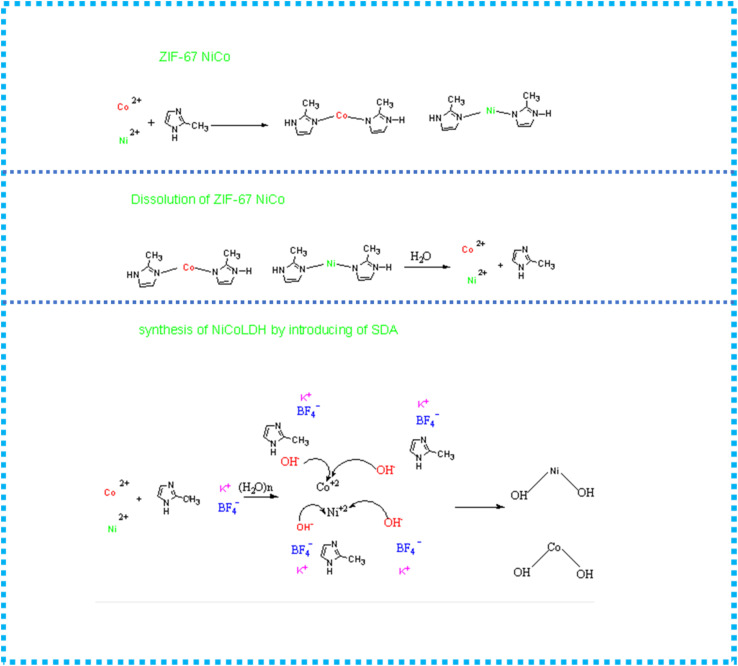
The formation reactions of ZIF-67 derived NiCo-LDH.

The XRD patterns of the synthesized nanocomposites shown in Fig. 1 indicate that the nanocomposites prepared with 24 h of stirring without a SDA consist of a pure phase with sharp and narrow diffraction peaks that match the reported bimetallic ZIF67-NiCo.^35^ When the SDA was introduced into the synthesized ZIF67-NiCo template in the presence of water, the sharp diffraction peaks of ZIF67-NiCo disappeared and the synthesized template converted to NiCo-LDH. The XRD patterns of NiCo-LDH, which is synthesized with various concentrations of SDA, were the same, as shown in Fig. 1. The XRD peaks for NiCo-LDH at 2*θ*: 11.34°, 23.07°, 33.88° and 59° can be indexed to (003), (006), (100), and (110), respectively and attributed to NiCo-LDH.^20,36^ To clearly explain the formation of ZIF-67-derived NiCo-LDH, the chemical reactions are illustrated in Scheme 1. It is observed that when using KBF_4_ as an SDA, the solvent contains OH^−^ groups, which is preferable for combination with Co and Ni to formed hydroxides. The function of KBF_4_ is likely to separate 2-MeIm from cobalt and nickel ions to prevent the formation of ZIF-67.

**Fig. 2:**
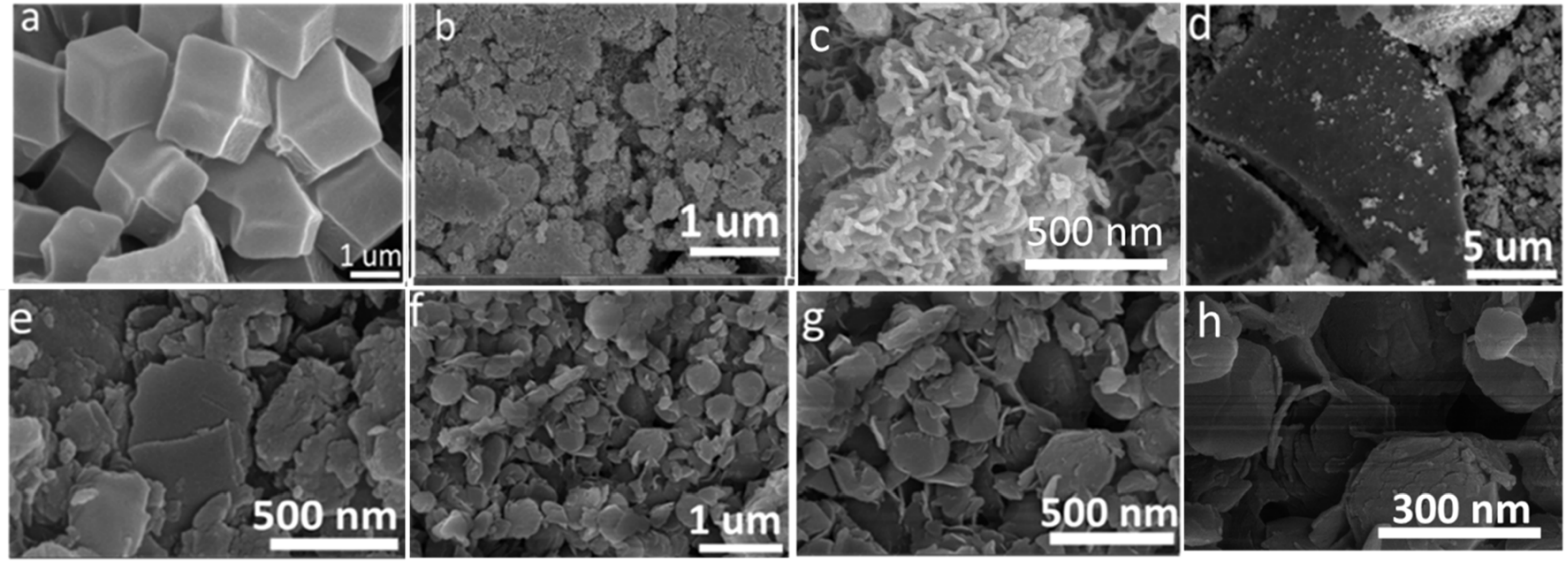
SEM images of (a) ZIF67-NiCo, (b and c) K3, (d and e) K2, and (f–h) K1.

**Fig. 3:**
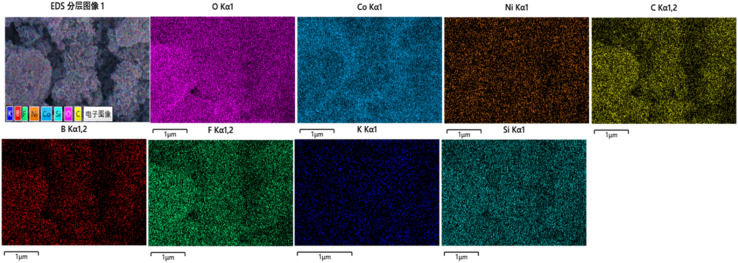
EDS mapping of NiCo-LDH (K3) nanocomposites.

The SEM images of bimetallic ZIF-67, which is synthesized from Ni and Co metals, are shown in Fig. 2a. The structure morphology of the ZIF67-NiCo is dodecahedral, and it possesses well-defined corners in accordance with the previously established ZIF-67 morphology.^37^ For the formation of different morphologies of the LDH, the SDA was used, as shown in Fig. 2. Fig. 2b and c show the structure morphology of the K3 nanocomposites that are synthesized with 0.35 g of SDA. The nanocomposites exhibit homogenous hollow nanosheets, which increase the specific surface area of the nanocomposites, and the porosity of the nanosheets offer a greater surface area for electrochemical reaction. The hollow-wrinkled nanosheets provide more free space to support the rapid movement of electrons.^38^ To systematically evaluate the effect of the SDA on the morphology of NiCo-LDH, the SEM images of K1 and K2 are also shown in Fig. 2. The SEM images of K2 are shown in Fig. 2d and e. The images show that the nanocomposites formed a nanosheet like morphology without any cavities. Moreover, when the SDA concentration is reduced to 0.15 g, the morphology of the nanocomposites changes to an agglomerate nano leaf like structure, as shown in Fig. 2f–h. For confirmation of the elemental composition, EDS was performed, as shown in Fig. 3. The EDS mapping of K3 shows that the O, Co, and Ni elements were uniformly distributed on the surface of the nanocomposites. Trace amounts of B, F, and K are present due to the use of KBF_4_ as a SDA.

**Fig. 4:**
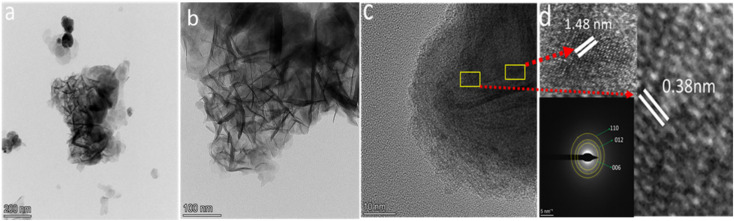
(a and b) TEM, and (c and d) HRTEM and SAED images of NiCo-LDH (K3).

To obtain more structure details TEM, HRTEM and SAED were performed for the NiCo-LDH, as shown in Fig. 4. The TEM image shows that the nanocomposites formed clear nanosheets with hollow cavities. SAED and HRTEM analysis were performed to investigate the crystallographic structure of the nanocomposites, as shown in Fig. 4c and d. In the HRTEM images the inter planer distances of 0.235 nm and 0.38 nm corresponding to the (012) and (006) planes of NiCo-LDH, respectively, are highlighted and are in agreement with the XRD results. The SAED pattern of K3 shows the (110), (012), and (006) planes of NiCo-LDH, which correspond to the XRD results, as shown in Fig. 4d. These findings confirm that the synthesized nanocomposite possesses NiCo-LDH characteristics.^39^

**Fig. 5:**
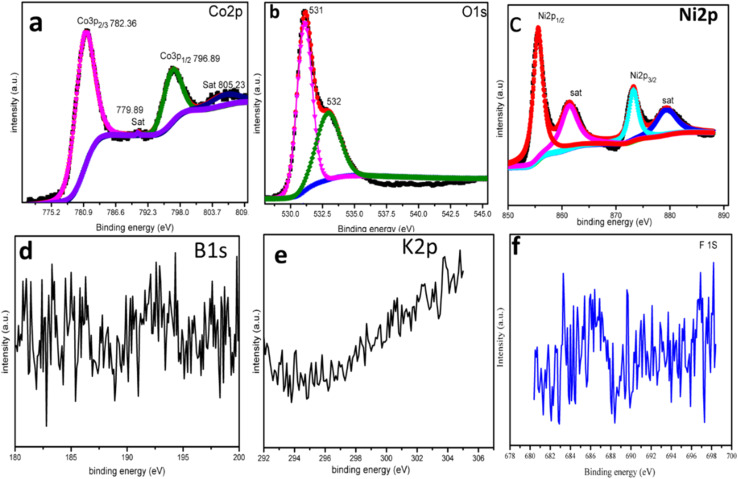
XPS of K3: (a) Co 2p, (b) O 1s, (c) Ni 2p, (d) B 1s, (e) K 2p, and (f) F 1s.

XPS was used to analyze the surface chemical oxidation states of the synthesized nanocomposites, and the results are shown in Fig. 5. The XPS spectrum of LDH shows three elements, Ni, Co, and O. In the high resolution spectrum of Co 2p, the two peaks located at 782 eV and 796 eV confirmed the presence of Co^2+^ and Co^3+^. The two satellite peaks seen at 779.89 eV and 805 eV correspond to the electronic states of Co 2p_2/3_ and Co 2p_1/2_, respectively, as shown in Fig. 5a .^40^ The Ni 2p peaks appear at 856 eV and 873 eV binding energies, corresponding to Ni 2p_3/2_ and Ni 2p_1/2_, respectively. The two satellite peaks seen at 879 eV and 861 eV indicate the presence of Ni^2+^ and Ni^3+^, as shown in Fig. 4c. The XPS peaks for O present at 531 eV and 532 eV, shown in Fig. 5d,^41^ are classified as corresponding to lattice oxygen (OII, ≈531.8 eV) and OH species or dissociated oxygen (OIII, ≈532.4 eV), respectively.^42^ The increase in intensity of the OII peak in the CoNi-LDH nanocomposites indicates a significant production of oxygen vacancies during the redox reaction. The formation of a large number of positive holes due to these defects is beneficial for electron transfer, resulting in remarkable specific capacitance.^42^ The B, K, and F signals are very weak and challenging to fit. However, the possible peaks were characterized and analyzed, as shown in Fig. 5d–f, respectively. The peaks of B, K, and F are present at binding energies of 196 eV, 292.5 eV, and 684 eV, respectively.^43,44^

**Fig. 6:**
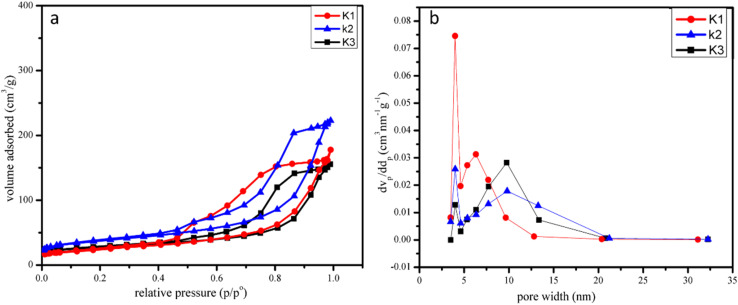
(a) Adsorption–desorption isotherms of K1, K2, and K3 nanocomposites; (b) pore size distribution of K1, K2 and K3 nanocomposites.

The specific surface area (SSA) plays a key role in the electrolyte adsorption and kinetics. For ion adsorption, mesoporous materials are particularly good, while the presence of macropores facilitates deeper inclusion to the material. Therefore, the presence of a variety of pore sizes in the material is essential for achieving optimal electrochemical behavior.^45^ Nitrogen adsorption–desorption isotherms used to investigate the SSA of the nanocomposites are shown in Fig. 6. The adsorption–desorption isotherm shows that the materials exhibit a type IV isotherm.^45^ The surface areas of the synthesized samples K3, K2, and K1 were 316 m^2^ g^−1^, 245 m^2^ g^−1^, and 185 m^2^ g^−1^, respectively, as shown in Fig. 6a. Barret–Joyner–Halenda (BJH) pore size distribution plots were studied, as shown in Fig. 6b. The pore size is from 1 to 30 nm, and the average pore volumes of the K1, K2, and K3 nanocomposites are 8, 12, and 25 nm, while the pore diameters of the nanocomposites are 9, 4, and 3.8 nm, respectively. The high porosity and large surface area of the synthesized materials facilitate the rapid transport of electrolyte ions, which leads to enhancement of the electrochemical performance.

### 3.1. Electrochemical analysis

**Fig. 7:**
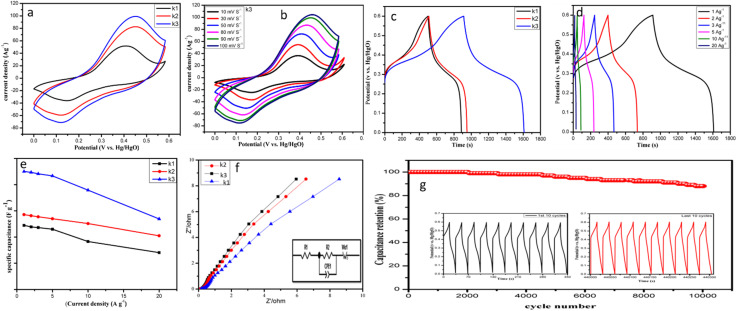
Electrochemical performance of the NiCo-LDH nanocomposite electrodes: (a) CV curves of the K3, K2 and K1 electrodes at 10 mV s^−1^; (b) CV curves of the K3 electrode; (c) GCD curves of the K3, K2 and K1 electrodes at 1 A g^−1^; (d) GCD curves of K3 at 1, 2, 3, 5, 10, and 20 A g^−1^; (e) specific capacitance *versus* charge–discharge current density of K3, K2 and K1; (f) Nyquist plots for the K3, K2 and K1 electrodes; and (g) cyclic stability at a current density of 20 A g^−1^ of the K3 electrode.

For the electrochemical study of the NiCo-LDH, a classical three-electrode setup was used to assess the charge storage capability. The tests including CV, GCD, and EIS were conducted in 2 M KOH electrolyte, and the NiCo-LDH was used as a working electrode, platinum served as a counter electrode, and Hg/HgO was used as a reference electrode. As can be seen from the CV curves (Fig. 7a), all samples exhibit obvious redox peaks, indicating contributions from transition metals such as Ni and Co.^46^ Oxidation and reduction reactions *i.e.*, Ni^2+^/Ni^3+^ and Co^2+^/Co^3+^ (and possibly Co^3+^/Co^4+^), produce a pair of redox peaks. The curve area of K3 is significantly larger than those of the other samples, demonstrating its high active site availability in the nanosheets and its contribution to the capacitance *via* the faradaic energy storage mechanism.^47^ The CV curves of K3 at a scan rate from 10 mV s^−1^ to 100 mV s^−1^ are displayed in Fig. 7b, while CV curves of K1 and K2 are also provided in the ESI† for comparison purposes (see Fig. S1†). The CV curves of all the samples consistently showed redox peaks at all current densities, indicating that the faradaic reaction is stable and reversible. The stable kinetics highlight the excellent properties of the NiCo-LDH porous network. The possible redox reactions of Ni and Co are described as follows.^48^

4 Ni(OH)_2_ + OH^−^ ↔ NiOOH + e^−^ + H_2_O

5 Co(OH)_2_ + OH^−^ ↔ CoOOH + e^−^ + H_2_O

6 CoOOH + OH^−^ ↔ CoO_2_ + e^−^ + H_2_O

Fig. 7c shows the GCD curves of K3, K2, and K1 at a current density of 1 A g^−1^, which reveal that all the electrodes exhibit nonlinear charge–discharge characteristics. This shows that the nanocomposite has pseudocapacitive behavior with a charge–discharge process, owing to the redox reactions involving Co(ii) to Co(iii) to Co(iv) and Ni(ii) to Ni(iii).^49^ Among all the electrodes, K3 exhibited the longest discharge duration, demonstrating high capacitive performance. The specific capacitances of K3, K2, and K1 are observed as 1171 F g^−1^, 743 F g^−1^, and 636 F g^−1^, respectively, at a current density of 1 A g^−1^, as displayed in Fig. 7c. The higher *C*_F_ of K3 is likely due to the larger surface area and the presence of active sites for electrolyte interaction. We also observed the GCD curves of K3 at different current densities from 1 A g^−1^ to 20 A g^−1^, as shown in Fig. 7d. The specific capacitances calculated for K3 are 1171, 1163, 1148, 1127, 986 and 700 F g^−1^ at current densities from 1 A g^−1^ to 20 A g^−1^, as shown in Fig. 7e. Comparative GCD curves of K2 and K1 are presented in the ESI (see Fig. S2).† The GCD curves of all the samples are non-liner and exhibit good symmetry at all current densities. At a wide scan rate, all the CV and GCD curves show excellent capability, which demonstrates the potential of the NiCo-LDH hybrid in supercapacitor applications with high power and energy density. The EIS results of K3, K2, and K1 shown in Fig. 7e show that the intersection of the *Z* axis and the impedance spectrum corresponds to the series resistance (*R*_s_), which represents the internal resistance and resistance of the electrode and electrolyte.^50^ The *R*_s_ values obtained for K3, K2, and K1 are 0.092 Ω, 0.11 Ω, and 0.15 Ω, respectively. In the low frequency range, the slope of the straight line represents the Warburg constant (*W*_0_), and electrodes with high slopes have lower resistances. It is clear that the K3 electrode has a significantly steeper slope than the other electrodes, indicating lower resistance. The constant phase element (CPE) represents the resistance at the electrode interface. The charge transfer resistance (*R*_ct_) values of K3, K2, and K1 are 0.199 Ω, 0.22 Ω, and 1.04 Ω, respectively. The lower *R*_ct_ value of K3 indicates that the electrode provides more active sites, thereby increasing the diffusion rate of ions, resulting in better electrochemical performance. Furthermore, the cyclic stability and specific retention were tested over 10 000 cycles of GCD testing at 20 A g^−1^. As shown in Fig. 7g, after 10 000 GCD cycles, excellent cycling stability of 88% was revealed. The charging–discharging curves of the device of the last 10 cycles (inset of Fig. 7g) are similar to those of the first 10 cycles, which indicates excellent cycling features. The capacitance maintained at 88% demonstrates good stability due to the large surface area and a hollow nanosheet like structure.^51–54^

**Fig. 8:**
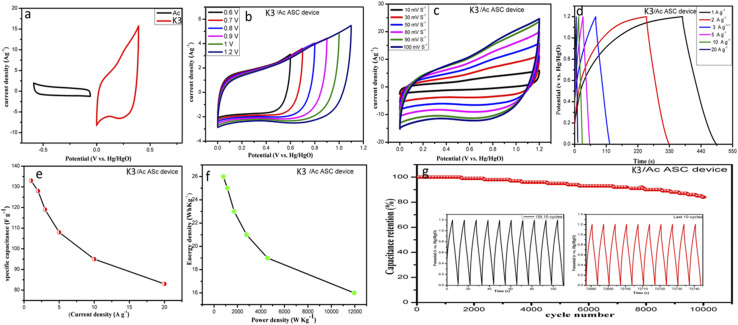
Electrochemical performance of the K3/AC ASC device: (a) CV curves of K3 and AC carbon electrodes at 40 m V s^−1^; (b) CV curves of the ASC device at 50 mV s^−1^ with different potential windows; (c) CV curves of the ASC device at scan rates ranging from 10 mV s^−1^ to 100 mV s^−1^; (d) GCD of the ASC device at current densities of 1 A g^−1^ to 20 A g^−1^; (e) specific capacitance *versus* charge–discharge current of the ASC device; (f) Ragone plot of the ASC device; (g) cyclic stability at a current density of 20 A g^−1^ for the ASC device.

To investigate the practical performance, K3/AC ASC devices were synthesized using 2 M KOH aqueous solution. The K3 was used as a positive electrode and AC was used as a negative electrode. The separation potential window for AC and NiCo-LDH at a scan rate of 50 mV s^−1^ is displayed in Fig. 8a. The CV curves indicate two different shapes, which proves that the electrode device possesses typical hybrid supercapacitor characteristics. CV curves of the K3/AC ASC device were recorded with an increasing voltage window at a constant scanning speed of 50 mV s^−1^ to investigate the optimum voltage rate, as shown in Fig. 8b. A voltage window was chosen between 0 and 1.2 V, where a significant increase in the current was observed. The CV curves of K3 at various current densities exhibited an almost rectangular shape, indicating an excellent voltage response, very close to the behavior of EDLC capacitors and battery capacitors, as can be seen in Fig. 8c. Meanwhile, the shape of the CV curves remains constant at all scanning rates, proving excellent reproducibility and good structure dynamics. The GCD curves of K3/AC ASC devices with different current densities are shown in Fig. 8d. All the GCD curves show quite high symmetry, which indicates good rate reversibility, and the energy storage device showed specific capacitances of 133 F g^−1^, 128 F g^−1^, 119 F g^−1^, 108 F g^−1^, 95 F g^−1^, and 83 F g^−1^ at current rates of 1 A g^−1^ to 20 A g^−1^, as shown in Fig. 8e. The specific power and energy consumption of the K3/AC ASC device were measured, showing a maximum energy density of 26 W h kg^−1^ with a power density of 804 W kg^−1^, as shown in Fig. 8f. Additionally, the device showed good structural stability, retaining 84% of its capacity after 10 000 charge–discharge cycles, as shown in Fig. 8g. In particular, the first 10 cycles were similar to the last 10 cycles, demonstrating remarkable cycling and good stability.

When we compare this work with work in the literature, we find that this work provides an excellent energy density, as shown in Table 1.

**Table 1:** Comparison of the electrochemical performance of the NiCo-LDH nanocomposite with related electrode materials

Materials	SDA	Specific capacitance, A g^−1^	Energy density, W h kg^−1^	Retention rate (%)/cycles	Reference
NiCo-LDH	NH_4_F	1527	15	90/5000	31
NiF_2_, CoF_2_	NH_4_F	735	24	96/6000	51
Ni(OH)_2_	NH_4_BF_4_	1593	15	97/6000	32
CoNi-N	NH_4_F	636	24	86/8000	52
NiCo-LDH	KBF_4_	1171	26	88/10 000	This work

## 4 Conclusion

We synthesized NiCo-LDH nanosheets from bimetallic ZIF67-NiCo *via* a facile and environmentally friendly method using a novel SDA, potassium tetrafluoride borate. The LDH nanosheets provide a greater surface area to support the electrochemical reaction, improving the charge transport pathway through the cavities present in the nanosheets. The synthesized LDH exhibits a high specific capacitance of 1171 F g^−1^ at 1 A g^−1^ and a cyclic stability of 88% after 10 000 charge–discharge cycles. The K3/AC ASC device exhibits an energy density of 26 W h kg^−1^ and a power density of 804 W kg^−1^, showing good cyclic stability of 84% after 10 000 charge cycles. This study reveals that ions can effectively overcome the interactions between the electrodes and the electrolyte interface, thereby improving performance. As a result, our findings substantiate the efficacy of aqueous-based energy storage systems in achieving superior energy storage capabilities, owing to their simple design and efficient use.

## Data availability

The data that support the findings of this study are available from the corresponding author upon reasonable request.

## Conflicts of interest

The authors declare that they have no known competing financial interests or personal relationships that could have appeared to influence the work reported in this paper.

## Supplementary Material

RA-015-D5RA01889G-s001

## References

[cit1] Gonçalves J. M. (2020). *et al.*, Trimetallic oxides/hydroxides as hybrid supercapacitor electrode materials: a review. J. Mater. Chem. A.

[cit2] Liu Y. (2019). *et al.*, Three-dimensional interconnected cobalt sulfide foam: Controllable synthesis and application in supercapacitor. Electrochim. Acta.

[cit3] Lei X. (2020). *et al.*, High capacity and energy density of Zn–Ni–Co–P nanowire arrays as an advanced electrode for aqueous asymmetric supercapacitor. ACS Appl. Mater. Interfaces.

[cit4] Sun J. (2020). *et al.*, Bundlelike CuCo2O4 microstructures assembled with ultrathin nanosheets as battery-type electrode materials for high-performance hybrid supercapacitors. ACS Appl. Energy Mater..

[cit5] Yang X. (2024). *et al.*, Tailoring ion-accessible pores of robust nitrogen heteroatomic carbon nanoparticles for high-capacity and long-life Zn-ion storage. J. Energy Storage.

[cit6] Li Y., A N. (2019). *et al.*, S dual doping strategy via electrospinning to prepare hierarchically porous carbon polyhedra embedded carbon nanofibers for flexible supercapacitors. J. Mater. Chem. A.

[cit7] Liu D. (2020). *et al.*, Core-shell Zn/Co MOFs derived Co3O4/CNTs as an efficient magnetic heterogeneous catalyst for persulfate activation and oxytetracycline degradation. Chem. Eng. J..

[cit8] Sun J., Xu C., Chen H. (2021). A review on the synthesis of CuCo2O4-based electrode materials and their applications in supercapacitors. J. Materiomics.

[cit9] Zhang D. (2024). *et al.*, Electrolyte additive strategies for safe and high-performance aqueous zinc-ion batteries: a mini-review. Energy Fuels.

[cit10] Liu Y. (2020). *et al.*, Co-ZIF derived porous NiCo-LDH nanosheets/N doped carbon foam for high-performance supercapacitor. Carbon.

[cit11] Zang Y. (2021). *et al.*, Polypyrrole nanotube-interconnected NiCo-LDH nanocages derived by ZIF-67 for supercapacitors. ACS Appl. Energy Mater..

[cit12] Yuksel R. (2020). *et al.*, Necklace-like nitrogen-doped tubular carbon 3D frameworks for electrochemical energy storage. Adv. Funct. Mater..

[cit13] Amali A. J., Sun J.-K., Xu Q. (2014). From assembled metal–organic framework nanoparticles to hierarchically porous carbon for electrochemical energy storage. Chem. Commun..

[cit14] Xiao Z. (2019). *et al.*, Construction of hollow cobalt–nickel phosphate nanocages through a controllable etching strategy for high supercapacitor performances. ACS Appl. Energy Mater..

[cit15] Hu H. (2015). *et al.*, Designed formation of Co3O4/NiCo2O4 double-shelled nanocages with enhanced pseudocapacitive and electrocatalytic properties. J. Am. Chem. Soc..

[cit16] Yu Z. (2017). *et al.*, Metal–organic-framework-derived yolk–shell-structured cobalt-based bimetallic oxide polyhedron with high activity for electrocatalytic oxygen evolution. ACS Appl. Mater. Interfaces.

[cit17] Xuan X. (2019). *et al.*, In-situ growth of hollow NiCo layered double hydroxide on carbon substrate for flexible supercapacitor. Electrochim. Acta.

[cit18] Lee G., Jang J. (2019). High-performance hybrid supercapacitors based on novel Co3O4/Co (OH) 2 hybrids synthesized with various-sized metal-organic framework templates. J. Power Sources.

[cit19] Hou S. (2020). *et al.*, Hollow dodecahedral Co3S4@ NiO derived from ZIF-67 for supercapacitor. Electrochim. Acta.

[cit20] Guan X. (2019). *et al.*, Facial design and synthesis of CoSx/Ni-Co LDH nanocages with rhombic dodecahedral structure for high-performance asymmetric supercapacitors. Chem. Eng. J..

[cit21] Elgendy A. (2020). *et al.*, Mesoporous Ni-Zn-Fe layered double hydroxide as an efficient binder-free electrode active material for high-performance supercapacitors. J. Power Sources.

[cit22] Ramachandran R. (2020). *et al.*, Construction of NiCo-layered double hydroxide microspheres from Ni-MOFs for high-performance asymmetric supercapacitors. ACS Appl. Energy Mater..

[cit23] Li X. (2019). *et al.*, (Ni, Co) Se2/NiCo-LDH core/shell structural electrode with the cactus-like (Ni, Co) Se2 core for asymmetric supercapacitors. Small.

[cit24] Yu M. (2017). *et al.*, Polyhedral-like NiMn-layered double hydroxide/porous carbon as electrode for enhanced electrochemical performance supercapacitors. Small.

[cit25] Zhao Y. (2020). *et al.*, Emerging CoMn-LDH@ MnO2 electrode materials assembled using nanosheets for flexible and foldable energy storage devices. J. Energy Chem..

[cit26] Wang H. (2023). *et al.*, Fe, Co-codoped layered double hydroxide nanosheet arrays derived from zeolitic imidazolate frameworks for high-performance aqueous hybrid supercapacitors and Zn-Ni batteries. J. Colloid Interface Sci..

[cit27] Bai X. (2017). *et al.*, Rational design of sandwiched Ni–Co layered double hydroxides hollow nanocages/graphene derived from metal–organic framework for sustainable energy storage. ACS Sustainable Chem. Eng..

[cit28] Xie W. (2024). *et al.*, A multifunctional platform based on ZIF-67 templated NiCo-LDH and Fe3O4 hollow spheres for photocatalytic CO2 conversion and supercapacitors. Surf. Interfaces.

[cit29] Alam N., Mokaya R. (2011). Super-micropore/small mesopore composite pillared silicate and aluminosilicate materials from crystalline layered silicate Na-RUB-18. Microporous Mesoporous Mater..

[cit30] Tang F., Li L., Chen D. (2012). Mesoporous silica nanoparticles: synthesis, biocompatibility and drug delivery. Adv. Mater..

[cit31] Lee P.-Y. (2023). *et al.*, Designing ZIF67 derivatives using ammonia-based fluorine complex as structure-directing agent for energy storage applications. J. Power Sources.

[cit32] Wang S.-C. (2023). *et al.*, Novel synthesis of ammonia borane fluoride induced ZIF67 derivatives using facile one-step solution process for energy storage. Mater. Today Chem..

[cit33] Chen Y. (2025). *et al.*, NH4+-Modulated Cathodic Interfacial Spatial Charge Redistribution for High-Performance Dual-Ion Capacitors. Nano-Micro Lett..

[cit34] Lee P.-Y. (2024). *et al.*, Concentration and reaction duration effects on synthesizing ZIF67 derivatives with NH4BF4 and NH4HF2 as efficient active material of energy storage device. J. Solid State Chem..

[cit35] Li M. (2019). *et al.*, CoNi-embedded nitrogen-enriched porous carbon framework for long-life lithium–sulfur batteries. J. Solid State Electrochem..

[cit36] Zhou Y. (2019). *et al.*, Unique 3D flower-on-sheet nanostructure of NiCo LDHs: Controllable microwave-assisted synthesis and its application for advanced supercapacitors. J. Alloys Compd..

[cit37] Wang Q. (2017). *et al.*, ZIF-67 derived amorphous CoNi2S4 nanocages with nanosheet arrays on the shell for a high-performance asymmetric supercapacitor. Chem. Eng. J..

[cit38] Tahir M. U. (2020). *et al.*, Room temperature and aqueous synthesis of bimetallic ZIF derived CoNi layered double hydroxides and their applications in asymmetric supercapacitors. J. Colloid Interface Sci..

[cit39] Zhong B. (2017). *et al.*, Large-scale fabrication and utilization of novel hexagonal/turbostratic composite boron nitride nanosheets. Mater. Des..

[cit40] Li B. (2023). *et al.*, Decoupling the roles of Ni and Co in anionic redox activity of Li-rich NMC cathodes. Nat. Mater..

[cit41] Wang M.-X. (2019). *et al.*, ZIF-67 derived Co 3 O 4/carbon aerogel composite for supercapacitor electrodes. New J. Chem..

[cit42] Wang Q. (2019). *et al.*, Redox tuning in crystalline and electronic structure of bimetal–organic frameworks derived cobalt/nickel boride/sulfide for boosted faradaic capacitance. Adv. Mater..

[cit43] Zhang H. , et al., MOF-Derived Hollow and Porous Co_3_O_4_ Nanocages for Superior Hybrid Supercapacitor Electrodes, 2021

[cit44] Chen J. (2018). *et al.*, Electronic-structure-dependent performance of single-site potassium catalysts for formaldehyde emission control. Ind. Eng. Chem. Res..

[cit45] Sundriyal S. (2023). *et al.*, Zeolitic Imidazole Framework Derived Cobalt Phosphide/Carbon Composite and Waste Paper Derived Porous Carbon for High-Performance Supercapattery. Adv. Mater. Interfaces.

[cit46] Tang Y. (2017). *et al.*, Engineering hollow polyhedrons structured from carbon-coated CoSe 2 nanospheres bridged by CNTs with boosted sodium storage performance. J. Mater. Chem. A.

[cit47] Cheng C. (2023). *et al.*, In Situ Growth of Nickel–Cobalt Metal Organic Frameworks Guided by a Nickel–Molybdenum Layered Double Hydroxide with Two-Dimensional Nanosheets Forming Flower-Like Struc-Tures for High-Performance Supercapacitors. Nanomaterials.

[cit48] Zhang C. (2024). *et al.*, Introduction of bimetallic oxide-modified carbon nanotubes for boosting the energy storage performance of NiCo-LDH based in-plane micro-supercapacitors on paper. Chem. Eng. J..

[cit49] Zhang F. (2017). *et al.*, Hierarchical flower-like nickel phenylphosphonate microspheres and their calcined derivatives for supercapacitor electrodes. J. Mater. Chem. A.

[cit50] Mei B.-A. (2018). *et al.*, Physical interpretations of Nyquist plots for EDLC electrodes and devices. J. Phys. Chem. C.

[cit51] Lee P.-Y., Lin L.-Y. (2022). Developing zeolitic imidazolate frameworks 67-derived fluorides using 2-methylimidazole and ammonia fluoride for energy storage and electrocatalysis. Energy.

[cit52] Lee P.-Y., Lin L.-Y. (2022). Investigating energy storage ability of ZIF67-derived perovskite fluoride via tuning ammonium fluoride amounts. J. Alloys Compd..

[cit53] Rehman Z. U. (2022). Synthesis and characterization of Ni nanoparticles via the microemulsion technique and its applications for energy storage devices. Materials.

[cit54] Ahmad S. (2024). *et al.*, A tremella-like in situ synthesis of ZIF-67Co (OH) F@ Co 3 O 4 on carbon cloth as an electrode material for supercapacitors. RSC Adv..

