# Supplementary material for: Structure-directed synthesis of bimetallic ZIF-67 LDH nanocomposites for high-performance supercapacitors
Source: RSC Adv. 2025 May 19;15(21):16667–76. doi: 10.1039/d5ra01889g (PMC12086822; doi:10.1039/d5ra01889g)
Supplement: RA-015-D5RA01889G-s001 [file RA-015-D5RA01889G-s001.pdf]

Supplementary Information (SI) for RSC Advances.
This journal is © The Royal Society of Chemistry 2025

Supporting information

Structure-directed synthesis of bimetallic ZIF-67 LDH nanocomposites for high-performance supercapacitors

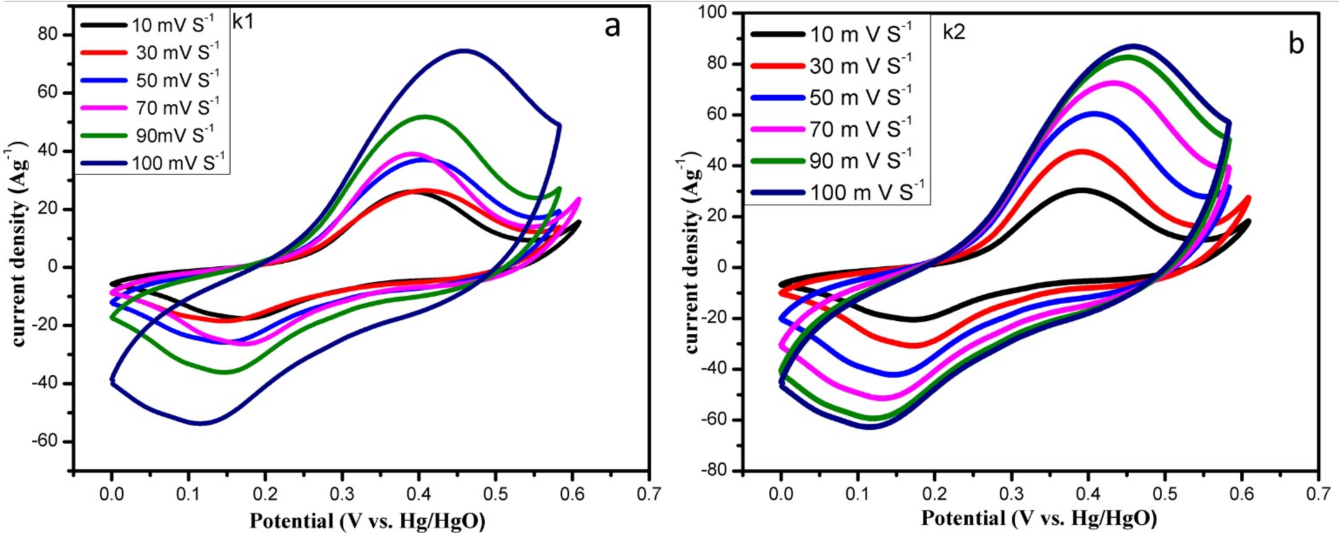

Figure. S1 (a) CV curves of K1 (b) CV curves of K2

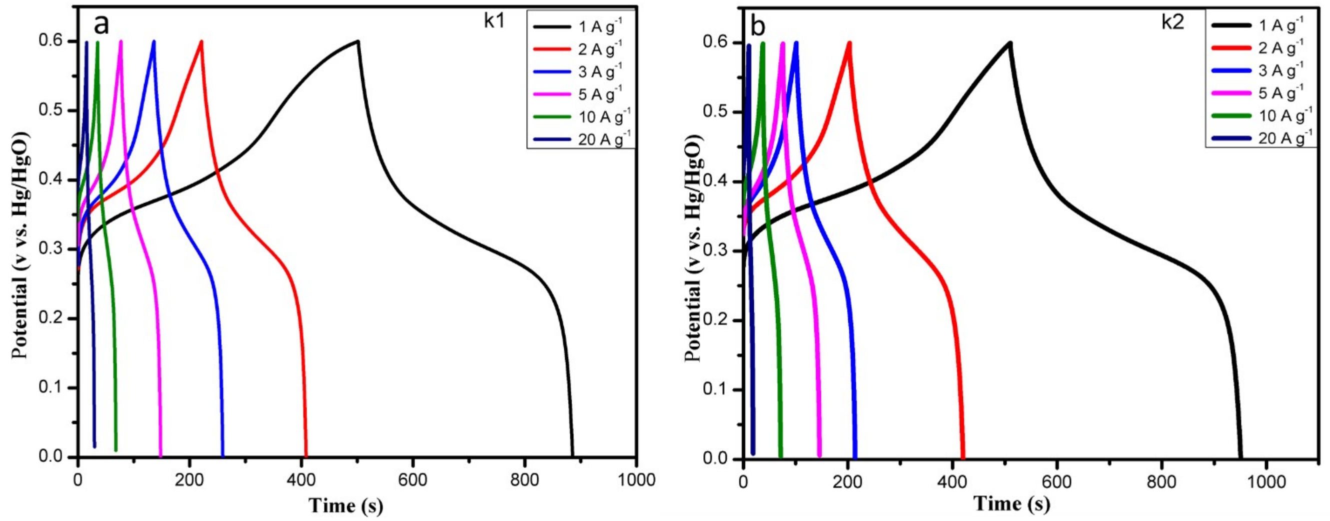

Figure. S2 (a) GCD curves of K1 (b) GCD curves of K2
